# Pan-Cancer Analysis of the Immunological Role of PDIA5: A Potential Target for Immunotherapy

**DOI:** 10.3389/fimmu.2022.881722

**Published:** 2022-08-08

**Authors:** Yu Chen, Jialin He, Rui Chen, Zeyu Wang, Ziyu Dai, Xisong Liang, Wantao Wu, Peng Luo, Jian Zhang, Yun Peng, Nan Zhang, Zaoqu Liu, Liyang Zhang, Hao Zhang, Quan Cheng

**Affiliations:** ^1^ Department of Neurosurgery, The First Affiliated Hospital of Zhengzhou University, Zhengzhou, China; ^2^ Department of Neurology, The Second Xiangya Hospital of Central South University, Changsha, China; ^3^ Department of Neurosurgery, Affiliated Nanhua Hospital, University of South China, Changsha, China; ^4^ Department of Neurosurgery, Xiangya Hospital, Central South University, Changsha, China; ^5^ National Clinical Research Center for Geriatric Disorders, Xiangya Hospital, Central South University, Changsha, China; ^6^ Department of Oncology, Xiangya Hospital, Central South University, Guangzhou, China; ^7^ Department of Oncology, Zhujiang Hospital, Southern Medical University, Changsha, China; ^8^ Department of Geriatrics, Xiangya Hospital, Central South University, Changsha, China; ^9^ Teaching and Research Section of Clinical Nursing, Xiangya Hospital of Central South University, Changsha, China; ^10^ One-third Lab, College of Bioinformatics Science and Technology, Harbin Medical University, Harbin, China; ^11^ Department of Interventional Radiology, The First Affiliated Hospital of Zhengzhou, Changsha, China; ^12^ Department of Neurosurgery, The Second Affiliated Hospital, Chongqing Medical University, Chongqing, China

**Keywords:** protein disulfide isomerase A5, pan-cancer analysis, cancer immunotherapy, bioinformatics, single-cell RNA sequencing

## Abstract

The aberrant protein disulfide isomerase A5 (PDIA5) expression was relevant to the poor prognosis of patients with human cancers. However, its relationship with the epigenetic and genetic alterations and its effect on tumor immunity is still lacking. In the present study, we comprehensively analyzed the immune infiltration role of PDIA5 in human cancers based on large-scale bioinformatics analyses and *in vitro* experiments. Obvious DNA methylation and moderate alteration frequency of PDIA5 were observed in human cancers. The expression level of PDIA5 was significantly correlated with infiltrated immune cells, immune pathways, and other immune signatures. We found that cancer cells and macrophages exhibited high PDIA5 expression in human cancers using the single-cell RNA sequencing analysis. We also demonstrated the interaction between PDIA5 and immune cells in glioblastoma multiforme (GBM). Multiplex immunofluorescence staining showed the upregulated expression level of PDIA5 and the increased number of M2 macrophage markers-CD163 positive cells in pan-cancer samples. Notably, PDIA5 silencing resulted in upregulated expression of PD-L1 and SPP1 in U251 cells. Silencing of PDIA5 in hepG2 cells, U251 cells, and PC3 cells contributed to a decline in their ability of proliferation, clone formation, and invasion and inhibited the migration of cocultured M2 macrophages. Additionally, PDIA5 also displayed predictive value in the immunotherapy response of both murine and human cancer cohorts. Overall, our findings indicated that PDIA5 might be a potential target for immunotherapies in cancers.

## Introduction

Since the critical role of the immunological landscape of tumor microenvironment (TME) in tumorigenesis has been increasingly recognized, cancer immunotherapies such as immune checkpoint inhibitors are promising (1, 2). Although these therapies have contributed to favorable clinical outcomes in multiple cancer types, some patients remain unresponsive to current immunotherapies (3, 4). Thus, identifying novel therapeutic targets and biomarkers of cancer is key to improving the efficacy of immunotherapy.

Protein disulfide isomerase (PDI) is a cluster of endoplasmic reticulum (ER) enzymes with both thiol-disulfide oxidoreductase and chaperone activities and plays a pivotal role in the modulation of proteostasis in the ER. PDI proteins mediate the formation, breakage, and rearrangement of protein disulfide bonds (5). PDI is also a molecular chaperone due to its aiding in the binding of misfolded protein for subsequent degradation (6). There are 21 members in the human PDI gene family; all of them possess at least one thioredoxin-like (TRX-like) domain (7). The TRX-like domain consists of an a-type (a or a′) domain and a b-type (b or b′) domain; the a-type domain is related to their function as oxidoreductase, whereas the b-type is responsible for the role of molecular chaperones (8).

More significantly, the involvement of PDI proteins in several disease states, particularly cancer, has also been gradually identified. PDI proteins impact the survival and apoptosis of cancer cells through a complex unfolded protein response (UPR) pathway, and the overexpression of PDI is related to the tumorigenesis, invasion, and metastasis of various cancers (9–13). Among those PDI proteins, PDIA3 mediates the induction of T cell tolerance, the differentiation of regulatory T cell, and the activation of macrophage/microglia, thereby contributing to the breakdown of immune surveillance and tumor cell invasion (14). PDIA4 is able to interact with activated platelet, enhance DNA repair machinery and induce drug-resistance in cancers (13). And high expression level of PDIA1 and PDIA6 correlate with the resistance to common chemotherapy drugs in cancer cells (15). In addition, PDIs inhibitors have already demonstrated positive anti-cancer effects in previous study. Altogether, PDI could serve as a potential therapeutic target and prognostic biomarker of cancers.

Protein disulfide isomerase A5 (PDIA5), also named protein disulfide isomerase-related (PDIR) protein, belongs to the human PDI family and has isomerase and chaperone activities (16, 17). A previous study proved that PDIA5 could cleave the disulfide bonds of UPR sensor ATF6, leading to its reduction and thereby inducing the UPR pathway (18). And the PDIA5/ATF6 signaling is correlated with the resistance to chemotherapy in leukemia cells, suggesting the pro-survival effect of PDIA5 in cancers (18). We have previously found that the expression level of PDIA5 was increased in multiple cancers compared to the normal tissues, with high PDIA5 expression correlating with unfavorable clinical outcomes (19). These observations imply that PDIA5 might be a therapeutic and prognostic pan-cancer biomarker.

Although the correlations between PDIA5 overexpression and immune infiltration in gliomas have been revealed (19), the impact of PDIA5 on the tumor immunity of human cancers have seldomly been explored systematically. In the present study, based on several well-known open databases and loss-of-function assays, we conducted a pan-cancer analysis to elaborate the PDIA5 profiles, including DNA methylation, genetic alterations, and its associations with immune infiltration and other signatures of interest in human cancers.

## Materials and Methods

### Data Acquisition and Preprocessing

We obtained pan-cancer samples of 33 cancer types from The Cancer Genome Atlas (TCGA) database. We downloaded the single-cell RNA sequencing (scRNA-seq) dataset of 13 cancer types from the Gene Expression Omnibus (GEO) database (https://www.ncbi.nlm.nih.gov/geo/) and BioProject dataset (https://www.ncbi.nlm.nih.gov/bioproject). The scRNA-seq datasets of bladder urothelial carcinoma (BLCA), cholangiocarcinoma (CHOL), colon adenocarcinoma (COAD), glioblastoma multiforme (GBM), head and neck squamous cell carcinoma (HNSC), liver hepatocellular carcinoma (LIHC), ovarian serous cystadenocarcinoma (OV), prostate adenocarcinoma (PRAD), skin cutaneous melanoma (SKCM), and stomach adenocarcinoma (STAD) were acquired from GSE145137, GSE125449, GSE81861, GSE138794, GSE103322, GSE125449, GSE118828, GSE137829, GSE72056, and GSE183904 in the GEO database, respectively. The scRNA-seq dataset of breast invasive carcinoma (BRCA) was downloaded from GSE75688 and GSE118389. The scRNA-seq dataset of kidney renal clear cell carcinoma (KIRC) was obtained from GSE121636 and GSE171306. In addition, the scRNA-seq dataset of lung adenocarcinoma (LUAD) was downloaded from the BioProject (#PRJNA591860).

### Bioinformatics Analysis

The GSCALite platform (http://bioinfo.life.hust.edu.cn/web/GSCALite/) was employed to analyze the methylation profiles, single nucleotide variation (SNV), and copy number variation (CNV) of PDI family genes (20). The role of PDI family genes in the classical oncogenic pathway was also investigated. Besides, the drug sensitivity and gene expression profile data of cancer cell lines in the Cancer Therapeutics Response Portal (CTRP) and Genomics of Drug Sensitivity in Cancer (GDSC) database were integrated for the drug sensitivity analysis. And the drug sensitivity analysis was performed using the Spearman correlation analysis; a positive value of the correlation coefficient represented drug resistance, while a negative one represented drug sensitivity.

The mutation landscape of PDIA5 in cancers was investigated using the cBioPortal for cancer genomics (http://www.cbioportal.org) (21).

The infiltration of immune cells and stromal cells in cancers was evaluated using the “estimate” package (22). The relationships between PDIA5 and infiltrated immune cells were explored by the Tumor IMmune Estimation Resource 2.0 (TIMER 2.0, http://timer.cistrome.org/) (23). The TISIDB website (http://cis.hku.hk/TISIDB/) examined the association between PDIA5 expression and immune subtypes of cancers (24). The correlation between PDIA5 and the gene ontology (GO) in terms of immune-related biological processes was analyzed *via* the gene set variation analysis (GSVA) (25). Correlation analysis was performed to assess the correlations between PDIA5 and mismatch repair (MMR) genes, microsatellite instability (MSI), tumor mutational burden (TMB), and DNA methyltransferases, immune checkpoint genes, as well as neoantigen as described previously (26).

The involvement of PDIA5 in human disease was identified by the OPENTARGET platform (https://www.targetvalidation.org/) (27), and the STRING database (https://string-db.org/cgi/input.pl) was used to predict the protein-protein interaction network of PDIA5. Besides, the immunotherapy response was predicted by the Tumor Immune Syngeneic MOuse database (http://tismo.cistrome.org), TIDE database (http://tide.dfci.harvard.edu), and ROC Plotter (http://www.rocplot.org/).

### Single-Cell RNA Sequencing Analysis

The integration of GSE75688 and GSE118389 for BRCA and GSE121636 and GSE171306 for KIRC were performed using the Anchors function of R package “Seurat” (28).

The scRNA-seq analysis was conducted as previously described (19). After scaling the data, we employed the principal component analysis (PCA) to reduce the dimension and then used the UMAP function for the visualization. The FindClusters function was performed for the cluster of cells. The R package “InferCNV” (29) and “CopyKAT” (30) were applied for the identification of malignant cells. And the annotation of stromal cells and immune cells was based on the specific markers. The Dimplot, FeaturePlot, and VlnPlot were used to visualize the expression of PDIA5 further. In addition, spatial transcriptomics was used to analyze the expression of PDIA5, CD68, and CD163 in BRCA (31).

Single-cell pseudotime trajectories analyses were performed using the R package “Monocle” (32); cells in the same segment of the trajectory were regarded as having the same “state”. GO enrichment analysis for the differentially expressed genes (DEGs) was further carried out. And the R package “CellChat” was used to perform the cell-cell interaction analysis (33).

### Multiplex Immunofluorescence Staining in Pan-Cancer Samples

We obtained the tissue microarray from the Outdo Biotech company (HOrg-C110PT-01, Shanghai, China) and the ethics was approved. Briefly, the paraffin sections of pan-cancer samples were deparaffinized and then were blocked with 3% H2O2 and 2% BSA after antigen retrieval. Anti-PDIA5 antibody (Rabbit, 1:100, Proteintech, China), anti-CD68 antibody (Rabbit, 1:3000, Servicebio, China), and anti-CD163 antibody (Rabbit, 1:3000, Proteintech, China) were sequentially applied, followed by horseradish peroxidase-conjugated secondary antibody (GB23301 and GB23303; Servicebio, China) incubation and tyramide signal amplification (TSA) (CY5-TSA, CY3-TSA, and FITC-TSA; Servicebio, China) incubation. And then, 4’,6-Diamidino2-phenylindole dihydrochloride (DAPI) was applied for the nuclei staining. The stained slides were visualized for multispectral images under the Pannoramic Scanner (3D HISTECH, Hungary). DAPI glows blue by UV excitation wavelength 330-380nm and emission wavelength 420nm in the fluorescence spectra. CY5, FITC, and CY3 glow pink, green, and red. The excitation wavelength was 608-648nm, 465-495nm, and 510-560nm, respectively, with an emission wavelength of 672-712nm, 515-555nm, and 590nm.

### Cell Lines and Cell Culture

Human LIHC cell line hepG2, GBM cell line U251, and PRAD cell line PC3 were cultured in Dulbecco’s modified eagle medium (DMEM) containing 10% fetal bovine serum (FBS) at 37°C in 5% CO_2_.

Human monocyte cell line THP-1 was cultured in 1640 complete medium containing 10% FBS at 37°C in 5% CO_2_. THP-1 cells cultured in 6-well plates (2.5 × 10^5^) were stimulated with 320nM phorbol 12-myristate 13-acetate (PMA; Sigma-Aldrich, St. Louis, MO, USA) and incubated at 37°C for 6h to induce M0 macrophages. For M2 macrophage polarization, M0 macrophages were treated with M2-polarizing reagents (20 ng/ml IL-4 and 20 ng/ml IL-13) and hatched at 37°C for 72h.

### Small Interfering RNA (siRNA) Knockdown of PDIA5

For siRNA knockdown, the siRNA target sequence of siRNA-PDIA5-1 and siRNA-PDIA5-2 was 5′- GCAAGAAGATGAAAGTTGA-3′and 5′- GACGGTTCTTGTTCCAGTA-3′, respectively. PDIA5 expression in HepG2, U251, and PC3 cells was silenced with PDIA5 siRNA using a siRNA transfection kit (Ribobio Co., Ltd, Guangzhou, China), according to the manufacturer’s instruction.

### Western Blotting Analysis

HepG2, U251, and PC3 cells were processed for western blotting. Immunoblot analyses were performed using the following primary antibodies: anti-PDIA5 (Rabbit, 1:2000, Proteintech, China), anti-secreted phosphoprotein 1 (SPP1; Rabbit, 1:1000 proteintech, China), anti-programmed death-ligand 1 (PD-L1; Mouse, 1:2000 proteintech, China) and anti-β-actin (Mouse, 1:5000, Proteintech, China). HRP goat anti-mouse IgG (Mouse, 1:5000, Proteintech, China) and HRP goat anti-rabbit IgG (Rabbit, 1:6000, Proteintech, China) were used as the secondary antibody. The proteins were visualized using an enhanced chemiluminescent (ECL) detection kit.

### Cell Counting Kit-8 (CCK8) Assay

HepG2, U251, and PC3 cells were seeded in a 96-well plate at the density of 1×10^4^ cells per well. The CCK8 reagent (Dojindo Molecular Technologies, Dojindo, Japan) was subsequently added per the manufacturer’s descriptions. Later, the si-NC and a si-PDIA5 group of hepG2, U251, and PC3 cells were cultured for 24h, 48h, and 72h, respectively. The absorbance was measured at 450nm after being incubated at 37°C in 5% CO2.

### Clone Formation Assay

The si-NC and si-PDIA5 groups of hepG2, U251, and PC3 cells were plated in 6-well plates (200 cells per well) and cultured at 37°C in 5% CO2 for 2 weeks. The colonies were fixed with 4% methanol (1 ml per well) for 15min and were then stained with 0.5% crystal violet for 30min at room temperature. The numbers of colonies larger than 2mm were counted.

### Transwell Assay for Invasion

Cell invasion assay was carried out with a 6-well Transwell (Corning, USA). The matrigel was diluted with DMEM at the ratio of 1:8 on the ice. Each upper chamber was added with 50uL diluted Matrigel and then incubated at 37°C for solidification. The si-NC and si-PDIA5 groups of hepG2, U251, and PC3 cells (5 × 10^5^) were seeded on the Matrigel in the upper chamber with a serum-free medium. The lower chamber was filled with a complete medium containing 10% FBS. After 24h, cells were fixed with 4% paraformaldehyde for 25min and stained with 0.1% crystal violet for 20min.

### Coculture Assay for the Migration of M2 Macrophage

Cell migration assay was performed with a 6-well Transwell (Corning, USA). The si-NC and si-PDIA5 groups of hepG2, U251, and PC3 cells (5 × 10^5^) were added to the lower chamber, respectively, and 5 × 10^5^ M2 macrophages were added to the upper chamber. After coculturing for 24h, M2 macrophages in the upper chamber were fixed with 4% paraformaldehyde and then stained with 0.1% crystal violet.

### Statistical Analysis

Statistical analyses were performed using the GSCALite platform and R software (version 3.6.1, https://www.r-project.org/). We used correlation analyses to measure the degree of correlation between certain variables. For data that followed a normal distribution, Student’s t-test and one-way analysis of variance (ANOVA) were performed to compare two groups and among more than two groups, respectively. When data didn't follow a normal distribution, we used Mann–Whitney test and Kruskal–Wallis test to compare two groups and among more than two groups, respectively. A p < 0.05 indicated statistical significance.

## Results

### Methylation Analysis and the Widespread Genetic Alterations of PDIA5 in Cancers

PDIA5 was involved in various human diseases, especially cancers, as shown in **Figure S1A**, and the interactions between PDIA5 and TXNDC12, PLRG1, PPP1R2, POLR2G, INTS8, SHPK, KIAA0391, HYOU1, as well as XBP1 were also depicted (**Figure S1B**). Our previous findings based on large-scale bioinformatic analysis suggested that PDIA5 expression was elevated in various cancers (19), but the relevant regulatory mechanism was still unclear.

Since DNA methylation is usually associated with downregulation of gene expression and directly affects the occurrence and progression of cancers (34), we then evaluated the methylation profiles of PDIA5 and other PDI family members (e.g., P4HB, PDIA2, PDIA3, PDIA4, and PDIA6) in human cancers from the TCGA dataset by using the GSCALite platform. Compared with matched normal tissues, the DNA methylation of PDIA5 was upregulated in LIHC, LUAD, BLCA, kidney renal papillary cell carcinoma (KIRP), thyroid carcinoma (THCA), and esophageal carcinoma (ESCA), whereas downregulated in KIRC, lung squamous cell carcinoma (LUSC), PRAD, HNSC, COAD and BRCA (**Figure S1C**). And the correlation analyses indicated negative correlations between the expression level of PDIA5 and DNA methylation in most cancer types (**Figure S1D**). However, the effects of DNA methylation on the cellular levels of PDIA5 in these types of cancers need to be investigated by wet experiments in the future.

In addition to DNA methylation, genetic alterations also impact gene expression. Therefore, we then analyzed the SNVs and CNVs of PDIA5 and other PDI family members in 33 human cancer types. The SNV rates of PDI genes ranged from 17% to 25% in 315 samples. The mutation rate of PDIA5 was 23%, the majority of the genetic aberrations were missense mutations, and this mutation was more common in uterine corpus endometrial carcinoma (UCEC) than in other cancer types (**Figure S2A**). All PDI genes were found to exhibit varying degrees of CNVs. More specifically, PDIA5 showed homozygous amplification in cervical squamous cell carcinoma and endocervical adenocarcinoma (CESC), LUSC, and OV (**Figure S2B**). And apparent heterozygous amplification and deletion of PDIA5 were also observed in multiple cancer types (**Figure S2C**). Further correlation analyses indicated positive correlations between the mRNA of PDIA5 and CNVs in most cancers (**Figure S2D**).

Meanwhile, we also employed the cBioPortal to assess the alteration frequency of PDIA5. LUSC displayed the highest alteration level, with the PDIA5 alteration frequency approaching 8% (**Figure S3A**). Mutation and amplification in PDIA5 genes accounted for the majority of alteration frequency in most cancer types (**Figure S3B**). A total of 102 mutation sites were found between amino acids 0 and 519, consisting of 80 missense mutations, 10 truncating mutations, 1 inframe mutations, 6 splice mutations, and 5 fusion mutations (**Figure S3C**). Among them, P386Rfs*26/7Afs*44 was the most frequent mutation site. Overall, the above results demonstrated the DNA methylation and alteration frequency of PDIA5 in cancers.

### PDIA5 Is Related to the Activity of Multiple Oncogenic Pathways and Drug Sensitivity in Human Cancers

Furthermore, we determined the role of PDIA5 and other PDI members in the classical signaling pathways of human cancer using the GSCALite platform. PDI proteins were related to the activation and inhibition of multiple oncogenic pathways. More specifically, PDIA5 expression was correlated with the activation and inhibition of the hormone androgen receptor pathway, the PI3K/Akt pathway, and the RTK pathway (**Figures S4A**, **S4B**).

We also investigated the correlations between PDIA5 expression profiles and drug sensitivity of cancer cell lines from the CTRP and GDSC databases. In the CTRP database, the high level of PDIA5 correlated with increasing drug resistance to PAC-1, teniposide, and tozasertib (**Figure S4C**). And the analysis results of the GDSC database indicated that high expression of PDIA5 could lead to the resistance to KIN001-102, PAC-1, and GSK1070916 (**Figure S4D**). PAC-1 and GSK1070916 are used to treat solid tumors; teniposide and tozasertib are always used to treat acute lymphocytic leukemia and chronic myeloid leukemia, respectively. However, the types of cancer for the treatment in which KIN001-102 is used remains unclear. Collectively, these findings suggested that the expression level of PDIA5 may impact the activity of multiple oncogenic pathways and participate in the resistance to some common chemotherapy drugs.

### High PDIA5 Expression Correlates With Immune Infiltration in Human Cancers

**Figure 1:**
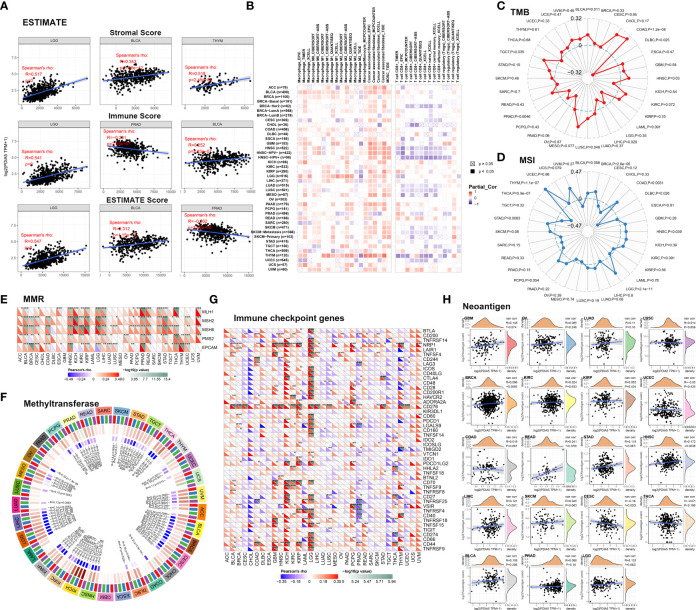
PDIA5 correlates with immune infiltration in human cancers. **(A)** The correlations between PDIA5 and stromal score, immune score, and ESTIMATE score. **(B)** The relationships between PDIA5 and immune cells. **(C)** The correlation analysis of PDIA5 and tumor mutational burden (TMB). **(D)** The correlation analysis regarding PDIA5 and microsatellite instability (MSI). **(E)** The relationships between the expression of PDIA5 and five mismatch repair (MMR) genes. **(F)** The correlation analysis regarding PDIA5 and four DNA methyltransferases. Red, blue, green, and purple represents DNMT1, DNMT2, DNMT3a, and DNMT3b. **(G)** The correlations between PDIA5 and immune checkpoint genes. **(H)** The correlation analysis of PDIA5 and the numbers of tumor neoantigens. The symbols “*”, “**”, and “***” mean p<0.05, p<0.01, and p<0.001, respectively.

The immune infiltration in TME promoting the progression of cancers has become increasingly distinguished (35), but the function of PDIA5 and its impact on the TME were not thoroughly investigated. In our study, PDIA5 exhibited strong correlations with a stromal score, immune score, and ESTIMATE score in various cancers. Among the 33 cancers, the top three cancers with the most remarkable correlation between PDIA5 expression and stromal score were lower-grade glioma (LGG), BLCA, and thymoma (THYM), the top three tumors whose PDIA5 expression was most notably associated with immune score were LGG, PRAD and BLCA. The top three relationships between PDIA5 and ESTIMATE score were LGG, BLCA and PRAD (**Figure 1A**).

Analyzing the role of PDIA5 in the infiltration of immune cells, we observed positive correlations between PDIA5 and macrophages, fibroblast, and myeloid-derived suppressor cells (MDSCs) in the majority of cancers. Conversely, there were negative correlations between PDIA5 and the abundance of CD8+ T cells in some cancer types, like BRCA, HNSC, LUSC, and testicular germ cell tumors (TGCT) (**Figure 1B**). These profiles revealed that high PDIA5 expression was involved in the immune infiltration process of human cancers.

### PDIA5 Is Relevant to Immune Subtypes and Immune Pathways in Human Cancers

The immune subtypes (36) showed that PDIA5 expression was relevant to different immune subtypes in GBM, LIHC, and PRAD (**Figure S5A**). The expression level of PDIA5 was higher in wound healing type (C1) than in lymphocyte depleted type (C4) and immunologically quiet type (C5) in GBM. The expression level of PDIA5 was highest in the TGF-β dominant type (C6) in LIHC. And in PRAD, the lymphocyte depleted type (C4) exhibited the highest expression level of PDIA5.

In addition, PDIA5 was closely related to the immune pathways in human cancers (**Figure S5B**). More significantly, the expression level of PDIA5 showed positive correlations with the T cells and macrophages-related immune pathways in BLCA, COAD, GBM, KIRC, KIRP, LGG, and rectum adenocarcinoma (READ), and negative correlations in CESC, LUSC, PRAD, and THCA. These results further demonstrated the participation of PDIA5 in the immune regulation of human cancers.

### PDIA5 Affects TMB, MSI, MMR, DNA Methyltransferase, Immune Checkpoint Genes, and Neoantigens in Human Cancers

TMB, MSI, MMR, and DNA methylation are several well-known signatures in the TME, being involved in the mutation and epigenetic alterations of the tumor and serving as independent predictors of immune checkpoint blockade (ICB) efficacy (37–40). We undertook to estimate the effect of PDIA5 expression on TME in-depth by conducting a correlation analysis between PDIA5 and these signatures. Significantly, we observed positive correlations between PDIA5 and TMB in BLCA, COAD, HNSC, LUSC, and TGCT, and negative correlation in diffuse large B-cell lymphoma (DLBC), LIHC, and PRAD (**Figure 1C**). For MSI, BRCA, COAD, HNSC, LGG, STAD, and THYM exhibited positive correlations, while DLBC and THCA exhibited negative correlations (**Figure 1D** **)**. There were positive correlations between the expression level of PDIA5 and MMR genes in most cancers except DLBC, GBM, acute myeloid leukemia (LAML), mesothelioma (MESO), sarcoma (SARC), and uterine carcinosarcoma (UCS) (**Figure 1E**). These results suggested that PDIA5 could maintain the viability of tumor cells through increasing DNA mismatch repair-related genes. In addition, PDIA5-high tumors were shown to exhibit a high level of DNA methyltransferases (**Figure 1F**), which indicated the latent ability of PDIA5 to modulate the epigenetic status of human cancers. Altogether, our analyses elucidated the correlations between PDIA5 and TMB, MSI, MMR, as well as DNA methyltransferase, demonstrating that PDIA5 may impact the antitumor immunity *via* modulating the mutation and epigenetic status in the TME.

Since studies have underlined the critical role of immune checkpoint genes in tumor immunotherapy (41), we investigate the association between PDIA5 and 47 known immune checkpoint genes. PDIA5 was notably related to immune checkpoint genes in multiple cancer types, such as GBM, kidney chromophobe (KICH), KIRP, LGG, pheochromocytoma, paraganglioma (PCPG), PRAD, and THYM (**Figure 1G**), indicating the potentiality of PDIA5 to regulate tumor immunity in these cancers.

Neoantigen refers to the mutated peptides expressed only by tumor cells, and neoantigen may contribute to the spontaneous antitumor immune responses and form a biomarker in cancer immunotherapy (42, 43). Positive correlations between PDIA5 and neoantigens were observed in BRCA and HNSC, while a negative correlation was found in CESC (**Figure 1H**). These results suggested a potential synergy of PDIA5 with neoantigens.

### Cancer Cells and Macrophages Exhibit High PDIA5 Expression in scRNA-Seq of Human Cancers

**Figure 2:**
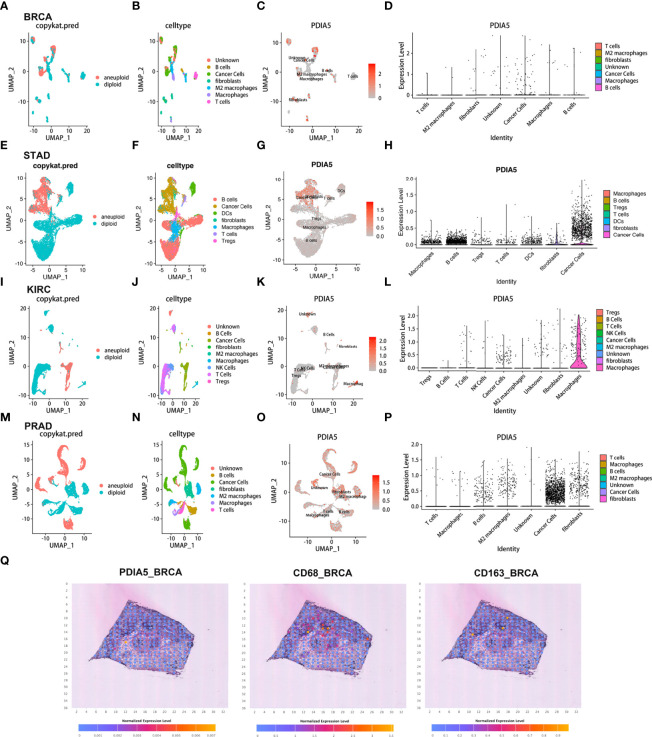
The scRNA-seq results of PDIA5 expression in pan-cancers. The definition of tumor cells in BRCA **(A)**, STAD **(E)**, KIRC **(I)**, and PRAD **(M)**. The cells were categorized into different clusters in BRCA **(B)**, STAD **(F)**, KIRC **(J)**, and PRAD **(N)**. The scatter plots depict the PDIA5 expression distribution of different cell clusters in BRCA **(C)**, STAD **(G)**, KIRC **(K)**, and PRAD **(O)**. The violin plot visualizes the distribution of PDIA5 expression of different cell clusters in BRCA **(D)**, STAD **(H)**, KIRC **(L)**, and PRAD **(P)**. **(Q)** The spatial transcriptomics of PDIA5, CD68, and CD163 in BRCA.

Using the scRNA-seq, we delineated the expression level and immune infiltration role of PDIA5 in BLCA, BRCA, CHOL, COAD, STAD, HNSC, KIRC, LIHC, LUAD, OV, PRAD, and SKCM (**Figures 2** and **Figures S6**, **S7** **)**. The cells were identified as aneuploid cells, and diploid cells were defined as cancer cells using the R package “CopyKAT” and aneuploid cells. And the R package “InferCNV” identified the cells as immune cells, malignant cells, and stromal cells (**Figures 2** and **Figures S6**, **S7** **)**. Different clusters of infiltrated immune cell types (e.g., macrophage, B cells, T cells, and so on) in each tumor sample were identified by immune cell-specific molecular markers (**Figure 2** and **Figures S6**, **S7**). The expression of PDIA5 in different clusters of cells in each tumor sample was visualized in **Figure 2** and **Figures S6**, **S7**, respectively. PDIA5 was abundantly expressed in cancer cells of all tumor samples. PDIA5 was enriched in various types of immune cells in different tumor samples. For example, macrophages and B cells exhibited high PDIA5 expression in BRCA, while cancer cells and fibroblasts showed high PDIA5 expression in STAD. Notably, PDIA5 was richly expressed in BRCA, KIRC, and PRAD macrophages. Furthermore, according to spatial transcriptomics, PDIA5 and macrophage markers (CD68 and CD163) were expressed at different levels in BRCA (**Figure 2Q**). Generally, these findings suggested that cancer cells and macrophages exhibited high PDIA5 expression in human cancers.

### PDIA5 Displays Interaction With Immune Cells in scRNA-Seq of GBM

**Figure 3:**
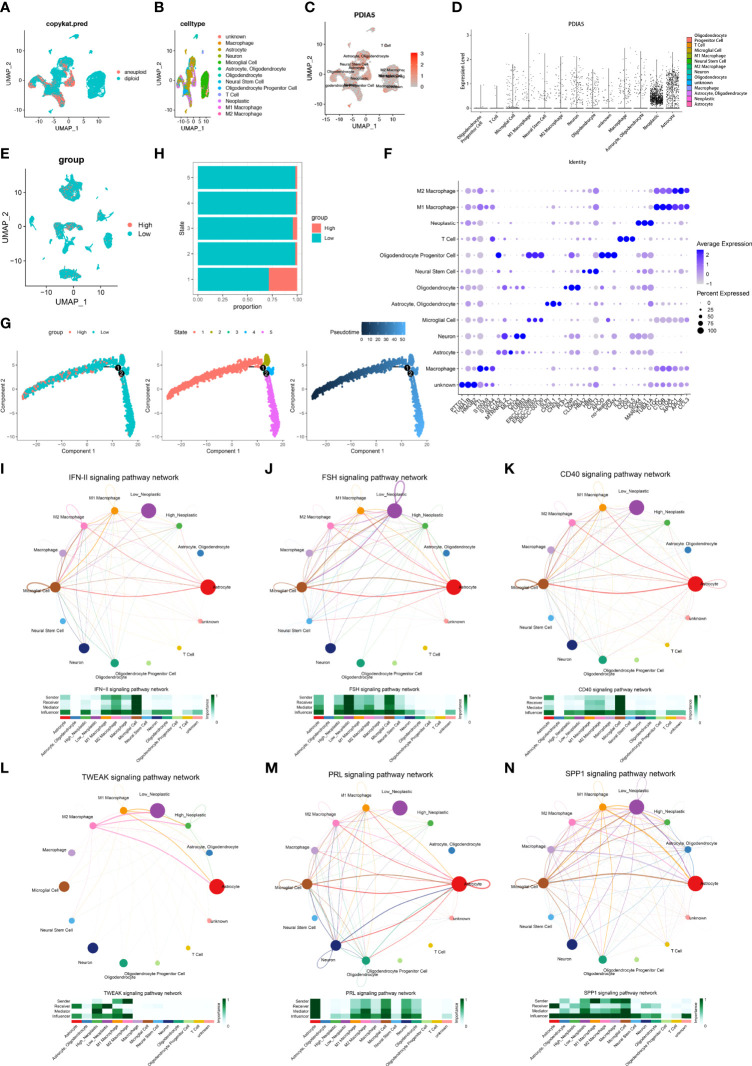
The scRNA-seq results of PDIA5 expression in GBM. **(A)** The definition of neoplastic cells in GBM. **(B)**The cells were categorized into 13 clusters in GBM. **(C)** The scatter plots visualize the PDIA5 expression distribution of different cell clusters in GBM. **(D)** The violin plot depicts the PDIA5 expression of varying cell clusters in GBM. **(E)** The visualization of the high and low expression levels of PDIA5 in all cell types. The red color represents those cells with high PDIA5 expression, while the blue represents low PDIA5 expression. **(F)** The differentially expressed genes (DEGs) among the 13 cell types in GBM. **(G)** The single-cell trajectory of neoplastic cells includes four branches. Cells are colored based on PDIA5 expression (left), state (middle), and pseudotime (right). **(H)** The proportion of cells with high and low PDIA5 levels in each state. **(I–N)** The signaling pathways involved in the correlations between neoplastic cells and immune cells in GBM: **(I)** IFN-II, **(J)** FSH, **(K)** CD40, **(L)** TWEAK, **(M)** PRL, and **(N)** SPP1 signaling pathway.

We next detected the characteristics of PDIA5 in GBM samples based on scRNA-Seq analysis. The definition of neoplastic cells in GBM was displayed in **Figure 3A**, and 13 cell types were further identified (**Figure 3B**). The expression of PDIA5 in different Cluster cells in GBM was depicted in **Figure 3C**. The Vlnplot further confirmed that PDIA5 was highly expressed in neoplastic cells, astrocytes, macrophages, neurons, and oligodendrocytes (**Figure 3D**). The high and low expression level of PDIA5 in all cell types was displayed in **Figure 3E**. Neoplastic cells with high PDIA5 expression were closely associated with the modulation of tumorigenic and immunogenic pathways (**Figure S8A**). The DEGs among the 13 cell types were visualized in **Figure 3F**. There is currently no survival/disease progression data in single-cell sequencing analysis that can be used for clinical research. Therefore, we cannot correlate the DEGs with the genetic background, stage of the disease, and outcome in terms of survival/disease progression.

Subsequently, we explored the single-cell pseudotime trajectory of PDIA5 in GBM samples, which grouped cells into 5 states, and the expression of PDIA5 decreased as the pseudotime increased (**Figure 3G**). PDIA5 was most expressed in state 1 compared with other states (**Figure 3H**). The top 100 downregulated and upregulated genes with the increase in pseudotime are shown in **Figure S8B**. GO enrichment analysis revealed that as pseudotime increased, upregulated genes were enriched in the synapse organization while downregulated genes were most involved in the extracellular matrix organization (**Figures S8C, D**).

The cellular communication of neoplastic cells with high and low PDIA5 expression was also explored using the R package “CellChat”. The roles of the identified 14 cell types in cellular communication were grouped into 4 types: receiver, sender, mediator, and influencer. The receiver and sender refer to the receiver and sender of the signaling pathway, respectively, and the mediator and influencer are the mediator and interferer of the signaling pathway, respectively. The cell patterns of the receiver and sender of the 14 cell types were classified into 3 distinct patterns (**Figures S9A, C**, respectively). The specific genes associated with the receiver and sender communication pattern of the 14 cell types also displayed 3 patterns (**Figures S9B, D**, respectively). The receiver and sender communication patterns of the 14 cell types were depicted as dot plots (**Figures S10A**, **B**, respectively). As shown in the river plot, astrocyte, high neoplastic cell, low neoplastic cell, and neural stem cell were correlated with the signaling pathways of the receiver in pattern 1 (**Figure S10C**). And astrocyte, oligodendrocyte, high neoplastic cell, low neoplastic cell, and neural stem cell demonstrated correlations with the signaling pathways of the sender in pattern 1 (**Figure S10D**).

The correlations between PDIA5 expression and specific signaling pathways were further illustrated. Overall, neoplastic cells with high expression of PDIA5 exhibited interaction with T cells *via* the IFN-II, CXCL, LT, CD40, TWEAK, MK, TRAIL, TGFβ, and FASLG signaling pathways (**Figures 3I–N** and **Figure S11**). Neoplastic cells with low expression of PDIA5 showed correlations with T cells through the CXCL, LT, FLT3, MK, TRAIL, PARs, TGFβ, and FASLG signaling pathways (**Figure S11**). Notably, neoplastic cells with high expression of PDIA5 showed interaction with macrophages *via* the IFN-II, FSH, CD40, TWEAK, PRL, and SPP1 signaling pathways (**Figures 3I–N**).

### PDIA5 Mediates the Invasion of Tumor Cells and Migration of M2 Macrophages

**Figure 4:**
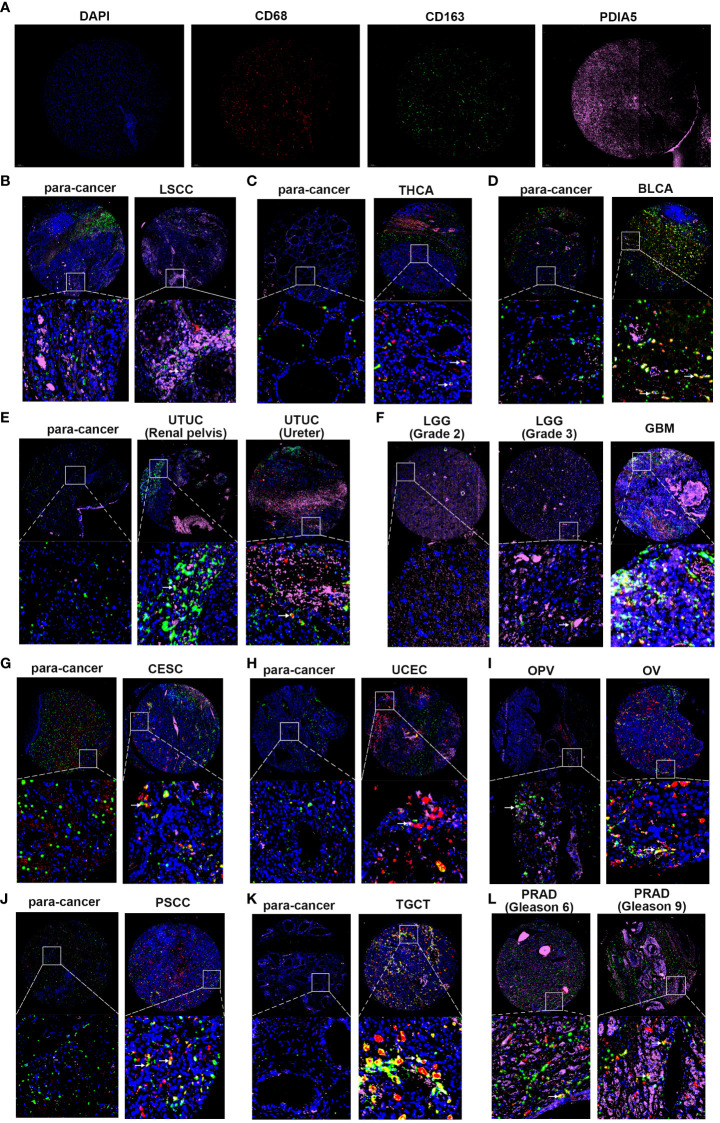
Multiplex immunofluorescence staining in pan-cancer samples and the corresponding para-cancerous tissues (para-cancer). **(A)** The representative image of DAPI, CD68, CD163, and PDIA5 staining in pan-cancer samples, respectively. Blue represents the DAPI-stained nucleus; red, green, and pink represent CD68-positive cells, CD163-positive cells, and PDIA5-positive cells. **(B)** laryngeal squamous cell carcinoma (LSCC), **(C)** thyroid carcinoma (THCA), **(D)** bladder urothelial carcinoma (BLCA), **(E)** urinary tract urothelial carcinoma (UTUC), **(F)** lower-grade glioma (LGG) and glioblastoma multiforme (GBM), **(G)** cervical squamous cell carcinoma and endocervical adenocarcinoma (CESC), **(H)** uterine corpus endometrial carcinoma (UCEC), **(I)** ovarian serous papillary cystadenocarcinoma (OPV) and ovarian serous cystadenocarcinoma (OV), **(J)** penis squamous cell carcinoma (PSCC), **(K)** testicular germ cell tumors (TGCT), **(L)** prostate adenocarcinoma (PRAD). The white arrow shows PDIA5 and CD163 double-positive cells, 10× amplification.

Using pan-cancer samples, we detected the PDIA5 expression in laryngeal squamous cell carcinoma (LSCC), THCA, BLCA, urinary tract urothelial carcinoma (UTUC), LGG, GBM, UCEC, CESC, ovarian serous papillary cystadenocarcinoma (OPV), OV, penis squamous cell carcinoma (PSCC), TGCT, and PRAD. As indicated in **Figure 4**, the immunofluorescence staining revealed that the numbers of PDIA5-positive cells were elevated in the LSCC, THCA, UTUC, CESC, UCEC, PSCC, and TGCT in comparison with the corresponding para-cancerous tissues. And the expression level of PDIA5 was increased as the malignance increased in several cancer types, as evidenced by the differences between LGG and GBM, OPV and OV, and the Gleason scores 6 and 9 of PRAD. In addition, the number of M2 macrophage markers-CD163 positive cells in most pan-cancer samples was significantly higher than that in para-cancerous tissues (**Figure 4**), suggesting the potential role of PDIA5 in the recruitment of M2 macrophage in the TME.

**Figure 5:**
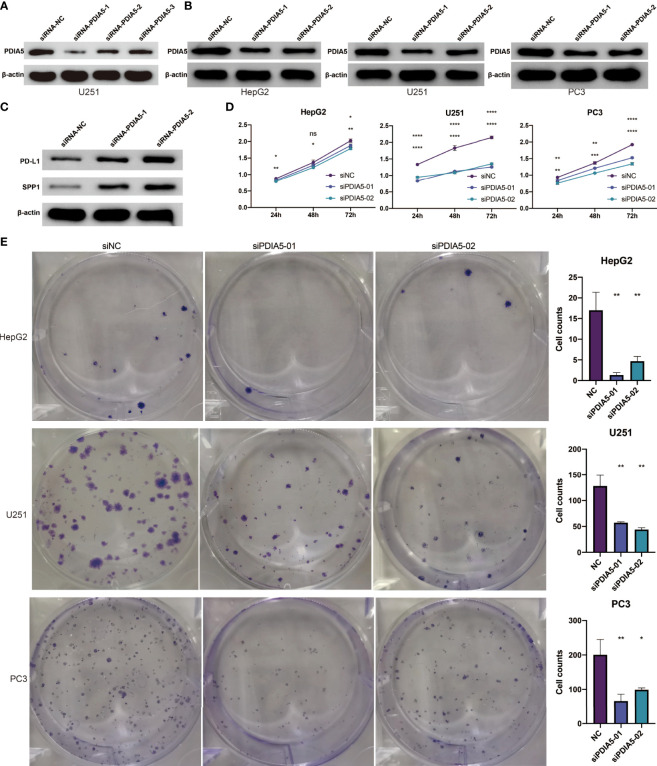
PDIA5 regulates the expression of PD-L1 and SPP1 in U251 cells and promotes the proliferation and clone formation of hepG2, U251, and PC3 cells. **(A)** The transduction results of different PDIA5 siRNA are verified using western blotting. **(B)** The protein expression level of PDIA5 in hepG2, U251, and PC3 cells after transfection with siRNA-PDIA5-1 and siRNA-PDIA5-2. **(C)** The protein expression level of PD-L1 and SPP1 in U251 cells after transfection with siRNA-PDIA5-1 and siRNA-PDIA5-2. **(D)** The cell proliferation of hepG2, U251, and PC3 cells after transfection of siRNA-PDIA5-1 and siRNA-PDIA5-2. **(E)** The clone formation of hepG2, U251, and PC3 cells after transfection of siRNA-PDIA5-1 and siRNA-PDIA5-2. Data are displayed as mean ± SD based on three independent experiments. *p < 0.05, **p < 0.01, ***p < 0.001, and ****p< 0.0001 compared with the normal control (NC) group. ns, no significance.

Subsequently, we explored the effect of PDIA5 on cancer cells and M2 macrophages *via* a loss-of-function assay. Firstly, a plasmid containing different PDIA5 siRNA sequences was constructed and transfected into human GBM cell line U251 cell. The western blotting analysis indicated that siRNA-PDIA5-1 and siRNA-PDIA5-2 contributed to a notable decrease in the protein level of PDIA5 compared with siRNA-NC (**Figure 5A**). Thereby, siRNA-PDIA5-1 and siRNA-PDIA5-2 were applied for further experiments.

As shown in **Figure 5B** and **Figure S12A–C**, siRNA-PDIA5-1 and siRNA-PDIA5-2 suppressed the expression level of PDIA5 protein in human LIHC cell line hepG2, GBM cell line U251, and PRAD cell line PC3 cells. To explore the effect of PDIA5 on immune pathways, we detected the expression level of PD-L1 and SPP1 in U251 cells after being transfected with PDIA5 siRNA. The protein expression levels of PD-L1 and SPP1 in the siRNA-PDIA5 group were notably higher than that of the siRNA Normal Control group (**Figure 5C** and **Figure S12D–E**), indicating that PDIA5 may exhibit adverse regulatory effects on the PD-L1 and SPP1 signaling pathway.

**Figure 6:**
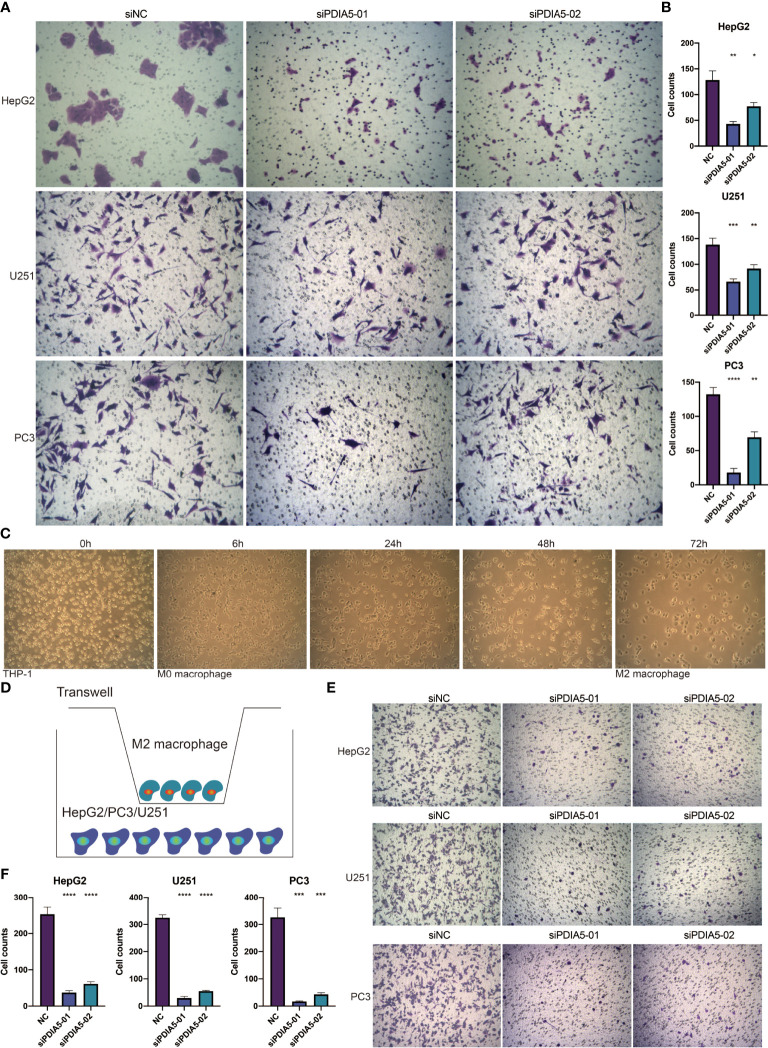
PDIA5 mediates the invasion of tumor cells and the migration of M2 macrophages. **(A, B)** The invasion of hepG2, U251, and PC3 cells after transfection of siRNA-PDIA5-1 and siRNA-PDIA5-2. **(C)** The induction process and morphology of M2 macrophages. **(D)** The schematic diagram illustrates the coculture of M2 macrophages and hepG2, U251, or PC3 cells. **(E, F)** The migration of M2 macrophages after being cocultured with hepG2, U251, and PC3 cells transfected with siRNA-PDIA5-1 and siRNA-PDIA5-2. Data are displayed as mean ± SD based on three independent experiments. *p < 0.05, **p < 0.01, ***p < 0.001, and ****p < 0.0001 compared with the normal control (NC) group.

Subsequently, we investigated the role of PDIA5 in the invasion of cancer cells and found that the cell proliferation, colony number, and cell invasion of hepG2, U251, and PC3 cells were impaired by the siRNA-PDIA5 (**Figures 5D, E**, **6A, B**).

Human THP-1 cells were induced to differentiate into M2 macrophages (**Figure 6C**), and we then used a Transwell device to co-culture M2 macrophages with hepG2, U251, or PC3 cells to investigate whether knockdown of PDIA5 in hepG2, U251, and PC3 cells could impact the migration ability of M2 macrophages (**Figure 6D**). Similarly, knockdown of PDIA5 in hepG2, U251, and PC3 cells also exhibited an inhibition effect on the migration ability of cocultured M2 macrophages (**Figures 6E, F**). Altogether, these findings demonstrated that PDIA5 could mediate the invasion of tumor cells and the migration of M2 macrophages.

### PDIA5 Exhibits Predictive Value in Immunotherapy Response

**Figure 7:**
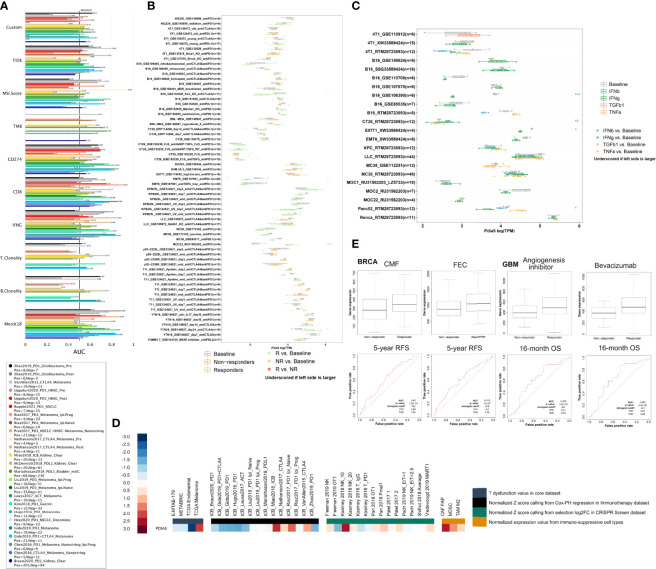
PDIA5 exhibits predictive value in immunotherapy response. **(A)** The PDIA5’s predictive power of response outcomes and overall survival (OS) in human immunotherapy cohorts. **(B)** The predictive value of PDIA5 in murine immunotherapy cohorts. **(C)** The expression levels of PDIA5 across cell lines between pre-and post-cytokine-treated groups. *p < 0.05, **p < 0.01, and ***p < 0.001 compared with the Baseline group. **(D)** The expression level of PDIA5 in different datasets. **(E)** The predictive value of PDIA5 in response to therapy in BRCA and GBM. RFS, relapse-free survival; OS, overall survival.

The biomarker correlation of PDIA5 was evaluated by comparing ng with standardized biomarkers. Their predictive power of response outcomes and overall survival (OS) of human immunotherapy cohorts was visualized in **Figure 7A**. Interestingly, we found that PDIA5 had an AUC of > 0.5 in 13 of the 25 immunotherapy cohorts, comparable to the MSI. Score. PDIA5 showed a higher predictive value than the TMB (AUC > 0.5 in 8 immunotherapy cohorts), T.Clonality (AUC > 0.5 in 9 immunotherapy cohorts), and B. Clonality (AUC > 0.5 in 7 immunotherapy cohorts), however, the predictive value of PDIA5 was lower than the TIDE, CD274, CD8, IFNG, and Merck18, which gave AUC values of > 0.5 in 18, 21, 18,17, and 18 immunotherapy cohorts, respectively.

PDIA5 could significantly predict immunotherapy response in 5 murine immunotherapy cohorts (**Figure 7B**); the resp; seemed to present high PDIA5 expression in 2 cohorts while presenting low PDIA5 expression in the remaining 3 cohorts. And the expression levels of PDIA5 across cell lines between pre-and post-cytokine-treated groups were summarized in **Figure 7C**. The IFNγ treatment decreased PDIA5 levels in 3 comparison groups, while the IFNβ treatment increased PDIA5 levels in 1 comparison group. Examining the expression level of PDIA5 in different datasets, we found that PDIA5 displayed upregulated expression levels in the CRISPR Screen dataset and immuno-suppressive cell types. In contrast, most of the expression levels of PDIA5 in the core and immunotherapy datasets were downregulated (**Figure 7D**). Further study regarding the response to therapy suggests that the responder exhibited high PDIA5 expression than the non-responder in BRCA and GBM (**Figure 7E**). Overall, these findings prove that PDIA5 displayed predictive value in the immunotherapy response of human cancers.

## Discussion

This study comprehensively investigated the DNA methylation, genetic alterations, and immune infiltration role of PDIA5 in human cancers based on large-scale bioinformatics analyses and *in vitro* assays. Our findings suggested that PDIA5 might be a potential target for cancer immunotherapy.

Accumulating evidence has demonstrated the correlation between DNA methylation and altered gene expression in cancers, in which DNA methylation could reduce mRNA level (44). Besides, CNVs could also modify gene mRNA expression (45). DNA methylation and CNVs statistically correlated with PDIA5 mRNA in our analysis. DNA methylation and CNVs showed a statistical correlation with PDIA5 mRNA, indicating that DNA methylation and CNVs of the PDIA5 gene might determine PDIA5 mRNA levels in tumors. Data regarding other proteins suggested that the DNA methylation and mutation frequency impacted its enzymatic activity and the disease progression and prognosis of patients. Still, the evidence in the field of PDIA5 remains largely unknown. Besides, the overall alteration rate of PDIA5 was relatively low across human cancers; therefore, the role of DNA methylation and PDIA5 alterations in its function and the occurrence and development of cancer warrants more in-depth exploration.

The links between PDIA5 and tumorigenesis in human cancers have not been uncovered previously; we found that PDIA5 expression may be responsible for the regulation of several famous tumor-associated pathways, including the hormone androgen receptor (46), PI3K/Akt (47) and RKT pathways (48). In addition, we also observed that high PDIA5 seemed to mediate the resistance to some common chemotherapy drugs in cancer cell lines. Since our study regarding the correlations between PDIA5 expression and drug sensitivity of cancer cell lines was predictive research based on existing databases, there is no available information linking the PDIA5 overexpression in these types of cancer and resistance to PAC-1, teniposide, tozasertib, KIN001-102, and GSK1070916 treatment. Accordingly, inspecting the mechanism underlying PDIA5 promotion of cancer development and drug resistance may contribute to the development of new therapeutic strategies.

The TME is complex and composed of malignant cells, endothelial cells, fibroblast, stromal and immune cells (49). The interplay between cancer cells and adaptive immune cells (T cells and B cells) and innate immune cells (macrophages, MDSCs, neutrophils, dendritic cells, and NK cells) results in tumor progression and metastasis. For example, the ability of cancer cells to acidify the microenvironment and deplete nutrients and oxygen could directly impair the function of CD8+ T cells (50). Moreover, tumor-associated macrophages (TAMs), MDSCs, fibroblasts, Treg cells, and endothelial cells in the TME could exert an immunosuppressive effect and promote T cell dysfunction by generating cytokines chemokines (51). Based on the correlation analyses between PDIA5 and infiltrated immune cells and other signatures of TME, the present study demonstrated the significant role of PDIA5 in cancer-related immunity.

Previous researchers have developed the ESTIMATE algorithm to evaluate stromal and immune cell infiltration in TME (22). These scores were reported to be negatively correlated with the clinical outcome of multiple human cancers. Interestingly, in our research, PDIA5 appeared to impact stromal score, immune score positively, and ESTIMATE score in some cancer types, such as BLCA, BRCA, COAD, and GBM, while exhibiting a negative effect on them in PRAD and THCA. This contradiction may be attributed to the distinct degree of immune cell infiltration in different cancers, and the expression of PDIA5 also varied widely among different cancer types.

In many cancers, our further analyses suggested that PDIA5 significantly correlated with immunosuppressive cells (including TAMs, fibroblast, and MDSCs). And high PDIA5 levels seemed to suppress the activity of CD8+ T cells and Treg cells in some tumor types. These observations indicated that PDIA5 might be closely related to immunosuppression and T cell dysfunction in human cancers. The subsequent correlation analyses about the immune subtypes and immune pathways further demonstrated the broad involvement of PDIA5 in tumor immunity regulation. As the specific mechanism regarding the effect of PDIA5 on immune cells in TME was largely unknown, more wet experiments were expected to be performed.

Our study revealed the close relationships between PDIA5 and immune checkpoint genes in multiple human cancers, strengthening the hypothesis that PDIA5 might enhance immunotherapy responses by synergizing with known immune checkpoint inhibitors in cancers. We also found that PDIA5 impacted neoantigens, TMB, MSI, MMR, and DNA methyltransferase in the TME. Collectively, these findings supported the possible intimate association between PDIA5 and TME and antitumor immunity.

In pan-cancer analysis, scRNA-seq has emerged as a potent tool to identify the cellular heterogeneity of TME within tumors (52, 53). Wang et al. identified several molecular differences between inflamed and noninflamed TMEs using scRNA-seq. These results have profound clinical implications as they may be helpful to the optimization of current combination immunotherapy (54). We also performed scRNA-seq to describe the landscape of PDIA5 in cell subpopulations within diverse cancer types. We found that PDIA5 was mainly enriched in cancer cells and macrophages, emphasizing the underlying role of PDIA5 in tumor immunity.

Pseudotime trajectories analysis is always applied to evaluate correlations between genes and specific cell clusters in human cancers as it can capture and dissect the transcriptional alteration in cells during tumor progression (32). And cell-to-cell communication analysis can predict how specific cells and signals coordinate for functions using network analysis and pattern recognition approaches (33, 55). In the present study, we conducted further research on the pseudotime trajectories analysis and cell-to-cell communication analysis of GBM. Neoplastic cells with high PDIA5 expression seemed to participate in the WNT, NOTCH, and MAPK signaling pathways. Moreover, we also identified several signaling pathways involved in the PDIA5 promoting macrophage infiltration and tumor progression. Nevertheless, these observations further highlighted the potentially significant role of PDIA5 in the progression and immune infiltration of human cancers.

To validate the relationships between PDAI5 and immune pathways in cancers, we investigated the effect of PDIA5 on the programmed death-1 (PD-1)/PD-L1 signaling pathway and SPP1 signaling pathway. The PD-1/PD-L1 pathway is responsible for the tumor immune tolerance and immune escape in human cancers, serving as one of the targets of ICB therapy (56). SPP1 can promote tumor invasion and inhibit the antitumor ability of immune cells through activating downstream signaling pathways (57). SPP1 overexpression was positively correlated with the severity of tumor malignancy and chemoresistance in multiple cancer types. Our study found that PDIA5 silencing in U251 cells contributed to upregulated expression of PD-L1 and SPP1, suggesting the possibly negative regulation effect of PDIA5 on the PD-1/PD-L1 signaling pathway and SPP1 signaling pathway in U251 cells. Given its various correlations with immune infiltration and complex biological regulation, the regulatory mechanism of PDIA5 in other immune pathways of human cancers need further investigation.

TAMs account for up to 50% of the cell population of the TME in some solid human malignancies, like breast cancer (58), gastric cancer (59) and hepatocellular carcinoma (60). Generally, high TAMs in the TME are relevant to poor prognosis in various tumors (61, 62). The lactate and cytokines produced by cancer cells facilitate the recruitment of blood-derived monocytes and promote their polarization toward an immunosuppressive M2-like state (63, 64). In addition to inhibiting the function of effector T cells, M2-like TAMs could also provide cancer cells with nutritional support, which further promotes the invasion and progression of cancer cells (65). Based on the evidence that PDIA5 showed a positive correlation with TAMs in correlation analyses, we further studied the effect of PDIA5 on tumor invasion and macrophage migration through multiplex immunofluorescence staining and loss-of-function assay. We observed that macrophages, especially M2 macrophages, were increased in multiple cancer types according to the multiplex immunofluorescence staining, suggesting the aggregation of M2 macrophages in the TME. Further loss-of-function experiments with hepG2 cells, U251 cell, PC3 cell, and M2 macrophages demonstrated that PDIA5 could mediate the proliferation and invasion of tumor cells and the migration of M2 macrophages.

We have previously concluded that PDIA5 could predict patients’ response to immunotherapies in gliomas as high PDIA5 displayed high anti-tumor immune activity (19), and the present study further revealed that PDIA5 exhibited predictive value in the immunotherapy response of both murine and human cancer cohorts, which may be related to its extensive effects on the TME.

Our study also has several limitations. First, validation in other cancer types or a large cohort is warranted in further research. Second, the underlying mechanisms by which PDIA5 takes part in immune regulation remain largely unknown; more loss-of-function assay is necessary to elucidate the specific role of PDIA5 in human cancers. Third, previous studies found that PDIA3 could mediate the induction of T cell tolerance, the differentiation of regulatory T cell, and the activation of macrophage/microglia in cancers. In the present study, our results demonstrated that PDIA5 was able to mediate the migration of M2 macrophages. Therefore, we think that both of PDIA5 and PDIA3 promote the progression of human cancers through affecting the infiltrated immune cells in tumor microenvironment, and the underlying mechanism remains further exploration.

In conclusion, our study provided comprehensive insights into the DNA methylation, genetic alterations, and immune infiltration role of PDIA5 across human cancers. These observations suggested that PDIA5 might serve as a potential target for the immunotherapy of human cancers.

## Data Availability Statement

The datasets generated for this study are available on request to the corresponding author/s.

## Author Contributions

RC, YC, and JH designed the study, interpreted data, and revised the manuscript. QC provided foundation support and supervised the study. HZ supervised the study. LZ revised the manuscript. ZW, ZD, XL, and HZ acquired and analyzed data. WW, PL, JZ, YP, ZL, and NZ drafted the manuscript and revised for submission quality. All authors read and approved the final manuscript.

## Funding

This work was supported by the National Natural Science Foundation of China (NO.82073893), Hunan Provincial Natural Science Foundation of China (NO.2022JJ20095), Hunan Provincial Health Committee Foundation of China (202204044869).

## Conflict of Interest

The authors declare that the research was conducted in the absence of any commercial or financial relationships that could be construed as a potential conflict of interest.

## Publisher’s Note

All claims expressed in this article are solely those of the authors and do not necessarily represent those of their affiliated organizations, or those of the publisher, the editors and the reviewers. Any product that may be evaluated in this article, or claim that may be made by its manufacturer, is not guaranteed or endorsed by the publisher.

## Supplementary Material

Supplementary Figure 1 The involvement of PDIA5 in human disease **(A)** and the prediction of the protein-protein interaction network of PDIA5 **(B)**. **(C)** The methylation difference of PDIA5 and other PDI family genes between tumor and corresponding normal tissues. **(D)** The relationships between methylation and the gene expression of PDIA5 and other PDI family genes.

Supplementary Figure 2 Pan-cancer genetic alterations of PDIA5 and other PDI genes. **(A)** The single nucleotide variation (SNV) frequency of PDIA5 and other PDI genes across 28 cancer types. **(B)** The frequency of homozygous amplification (left) and deletion (right) of PDIA5 and other PDI genes across cancer types. **(C)** The frequency of heterozygous amplification (left) and deletion (right) of PDIA5 and other PDI genes across cancer types. **(D)** The correlation analysis between copy number variations (CNVs) and mRNA expression of PDIA5.

Supplementary Figure 3 The mutation landscape of PDIA5. **(A)** The mutation frequency of PDIA5 in various cancer using cBioPortal database. **(B)** The general mutation count of PDIA5 in multiple cancers. **(C)** The mutation diagram of PDIA5 across protein domains.

Supplementary Figure 4 The pathway analysis and drug sensitivity of PDIA5 in human cancers. **(A, B)** The pie chart and network diagram demonstrating the correlations between PDIA5 and cancer pathways. **(C, D)** The correlation analysis of PDIA5 and IC50 level for each drug in cancer cell lines from the CTRP and GDSC databases.

Supplementary Figure 5 PDIA5 is relevant to immune subtypes and immune pathways in human cancers. **(A)** The relationships between PDIA5 and immune subtypes in GBM, liver hepatocellular carcinoma (LIHC), and PRAD. C1 to C6 represent wound healing, IFN-γ dominant, inflammatory, lymphocyte depleted, immunologically quiet, and TGF-β dominant, respectively. **(B)** The correlations between PDIA5 and immunity pathways.

Supplementary Figure 6 The scRNA-seq results of PDIA5 expression in pan-cancers. The definition of tumor cells in BLCA **(A)**, CHOL **(E)**, COAD **(I)**, and HNSC **(M)**. The cells were categorized into different clusters in BLCA **(B)**, CHOL **(F)**, COAD **(J)**, and HNSC **(N)**. The scatter plots depicting the PDIA5 expression distribution of different cell clusters in BLCA **(C)**, CHOL **(G)**, COAD **(K)**, and HNSC **(O)**. The violin plot visualizing the distribution of PDIA5 expression of different cell clusters in BLCA **(D)**, CHOL **(H)**, COAD **(L)**, and HNSC **(P)**.

Supplementary Figure 7 The scRNA-seq results of PDIA5 expression in pan-cancers. The definition of tumor cells in LIHC **(A)**, LUAD **(E)**, OV **(I)** and SKCM **(M)**. The cells were categorized into different clusters in LIHC **(B)**, LUAD **(F)**, OV **(J)** and SKCM **(N)**. The scatter plots depicting the PDIA5 expression distribution of different cell clusters in LIHC **(C)**, LUAD **(G)**, OV **(K)** and SKCM **(O)**. The violin plot visualizing the distribution of PDIA5 expression of different cell clusters in LIHC **(D)**, LUAD **(H)**, OV **(L)** and SKCM **(P)**.

Supplementary Figure 8 The scRNA-seq results of PDIA5 expression in GBM. **(A)** The correlation analysis between neoplastic cells with high or low PDIA5 expression and the biological process pathways of GO. **(B)** The top 100 downregulated and upregulated genes with the increase in pseudotime. **(C)** GO enrichment analysis of the upregulated genes in pseudotime trajectory analysis. **(D)** GO enrichment analysis of the downregulated genes in pseudotime trajectory analysis.

Supplementary Figure 9 The cellular communication of the 14 cell types in GBM. **(A, B)** The cell patterns and communication patterns with regard to the receiver of the 14 cell types. **(C, D)** The cell patterns and communication patterns with regard to the sender of the 14 cell types.

Supplementary Figure 10 The cellular communication of the 14 cell types in GBM. **(A, B)** The dot plots visualizing the receiver communication and sender communication patterns of the 14 cell types in GBM. **(C, D)** The river plot depicting the receiver communication and sender communication patterns of the 14 cell types in GBM.

Supplementary Figure 11 The signaling pathways involved in the correlations between neoplastic cells and T cells in GBM. **(A)** CXCL, **(B)** LT, **(C)** MK, **(D)** TRAIL, **(E)** TGFβ, **(F)** FASLG, **(G)** FLT3, and **(H)** PARs signaling pathway.

Supplementary Figure 12 PDIA5 regulates the expression of PD-L1 and SPP1 in U251 cells. **(A–C)** The bar graph showing the protein expression level of PDIA5 in hepG2, U251, and PC3 cells after transfection with siRNA-PDIA5-1 and siRNA-PDIA5-2, respectively. **(D)** The bar graph displaying the protein expression level of PD-L1 in U251 cells after transfection with siRNA-PDIA5-1 and siRNA-PDIA5-2. **(E)** The bar graph showing the protein expression level of SPP1 in U251 cells after transfection with siRNA-PDIA5-1 and siRNA-PDIA5-2. Data are displayed as mean ± SD based on three independent experiments. ∗p < 0.05, ∗∗p < 0.01.

## Abbreviations

TME, tumor microenvironment; PDI, Protein disulfide isomerase; ER, endoplasmic reticulum; TRX-like, thioredoxin-like; UPR, unfolded protein response; PDIA5, Protein disulfide isomerase A5; PDIR, protein disulfide isomerase-related; TCGA, The Cancer Genome Atlas; scRNA-seq, single-cell RNA sequencing; GEO, Gene Expression Omnibus; BLCA, bladder urothelial carcinoma; CHOL, cholangiocarcinoma; COAD, colon adenocarcinoma; GBM, glioblastoma multiforme; HNSC, head and neck squamous cell carcinoma; LIHC, liver hepatocellular carcinoma; OV, ovarian serous cystadenocarcinoma; PRAD, prostate adenocarcinoma; SKCM, skin cutaneous melanoma; BRCA, breast invasive carcinoma; KIRC, kidney renal clear cell carcinoma; STAD, stomach adenocarcinoma; LUAD, lung adenocarcinoma; SNV, single nucleotide variation; CNV, copy number variation; CTRP, Cancer Therapeutics Response Portal; GDSC, Genomics of Drug Sensitivity in Cancer; GO, gene oncology; GSVA, gene set variation analysis; MMR, mismatch repair; MSI, microsatellite instability; TMB, tumor mutational burden; DEGS, differentially expressed genes; TSA, tyramide signal amplification; DMEM, Dulbecco’s modified eagle medium; FBS, fetal bovine serum; CCK8, Cell Counting Kit-8; ANOVA, one-way analysis of variance; LSCC, laryngeal squamous cell carcinoma; THCA, thyroid carcinoma; UTUC, urinary tract urothelial carcinoma; LGG, lower grade glioma; UCEC, uterine corpus endometrial carcinoma; CESC, cervical squamous cell carcinoma and endocervical adenocarcinoma; OPV, ovarian serous papillary cystadenocarcinoma; PSCC, penis squamous cell carcinoma; TGCT, testicular germ cell tumors; KIRP, kidney renal papillary cell carcinoma; ESCA, esophageal carcinoma; LUSC, lung squamous cell carcinoma; THYM, thymoma; MDSCs, myeloid-derived suppressor cells; Treg cells, regulatory T cells; READ, rectum adenocarcinoma; ICB, immune checkpoint blockade; DLBC, diffuse large B-cell lymphoma; MESO, mesothelioma; SARC, sarcoma; UCS, uterine carcinosarcoma; KICH, kidney chromophobe; PCPG, pheochromocytoma and paraganglioma; OS, overall survival; TAMs, tumor-associated macrophages; SPP1, secreted phosphoprotein 1; PD-L1, programmed death-ligand 1; PD-1, programmed death.

## Glossary

**Table:**

TME	tumor microenvironment
PDI	Protein disulfide isomerase
ER	endoplasmic reticulum
TRX-like	thioredoxin-like
UPR	unfolded protein response
PDIA5	Protein disulfide isomerase A5
PDIR	protein disulfide isomerase-related
TCGA	The Cancer Genome Atlas
scRNA-seq	single-cell RNA sequencing
GEO	Gene Expression Omnibus
BLCA	bladder urothelial carcinoma
CHOL	cholangiocarcinoma
COAD	colon adenocarcinoma
GBM	glioblastoma multiforme
HNSC	head and neck squamous cell carcinoma
LIHC	liver hepatocellular carcinoma
OV	ovarian serous cystadenocarcinoma
PRAD	prostate adenocarcinoma
SKCM	skin cutaneous melanoma
BRCA	breast invasive carcinoma
KIRC	kidney renal clear cell carcinoma
STAD	stomach adenocarcinoma
LUAD	lung adenocarcinoma
SNV	single nucleotide variation
CNV	copy number variation
CTRP	Cancer Therapeutics Response Portal
GDSC	Genomics of Drug Sensitivity in Cancer
GO	gene oncology
GSVA	gene set variation analysis
MMR	mismatch repair
MSI	microsatellite instability
TMB	tumor mutational burden
DEGS	differentially expressed genes
TSA	tyramide signal amplification
DMEM	Dulbecco’s modified eagle medium
FBS	fetal bovine serum
CCK8	Cell Counting Kit-8
ANOVA	one-way analysis of variance
LSCC	laryngeal squamous cell carcinoma
THCA	thyroid carcinoma
UTUC	urinary tract urothelial carcinoma
LGG	lower grade glioma
UCEC	uterine corpus endometrial carcinoma
CESC	cervical squamous cell carcinoma and endocervical adenocarcinoma
OPV	ovarian serous papillary cystadenocarcinoma
PSCC	penis squamous cell carcinoma
TGCT	testicular germ cell tumors
KIRP	kidney renal papillary cell carcinoma
ESCA	esophageal carcinoma
LUSC	lung squamous cell carcinoma
THYM	thymoma
MDSCs	myeloid-derived suppressor cells
Treg cells	regulatory T cells
READ	rectum adenocarcinoma
ICB	immune checkpoint blockade
DLBC	diffuse large B-cell lymphoma
MESO	mesothelioma
SARC	sarcoma
UCS	uterine carcinosarcoma
KICH	kidney chromophobe
PCPG	pheochromocytoma and paraganglioma
OS	overall survival
TAMs	tumor-associated macrophages
SPP1	secreted phosphoprotein 1
PD-L1	programmed death-ligand 1
PD-1	programmed death
