# Supplementary figures and images for: Pan-Cancer Analysis of the Immunological Role of PDIA5: A Potential Target for Immunotherapy
Source: Front Immunol. 2022 Aug 8;13:881722. doi: 10.3389/fimmu.2022.881722 (PMC9393377; doi:10.3389/fimmu.2022.881722)

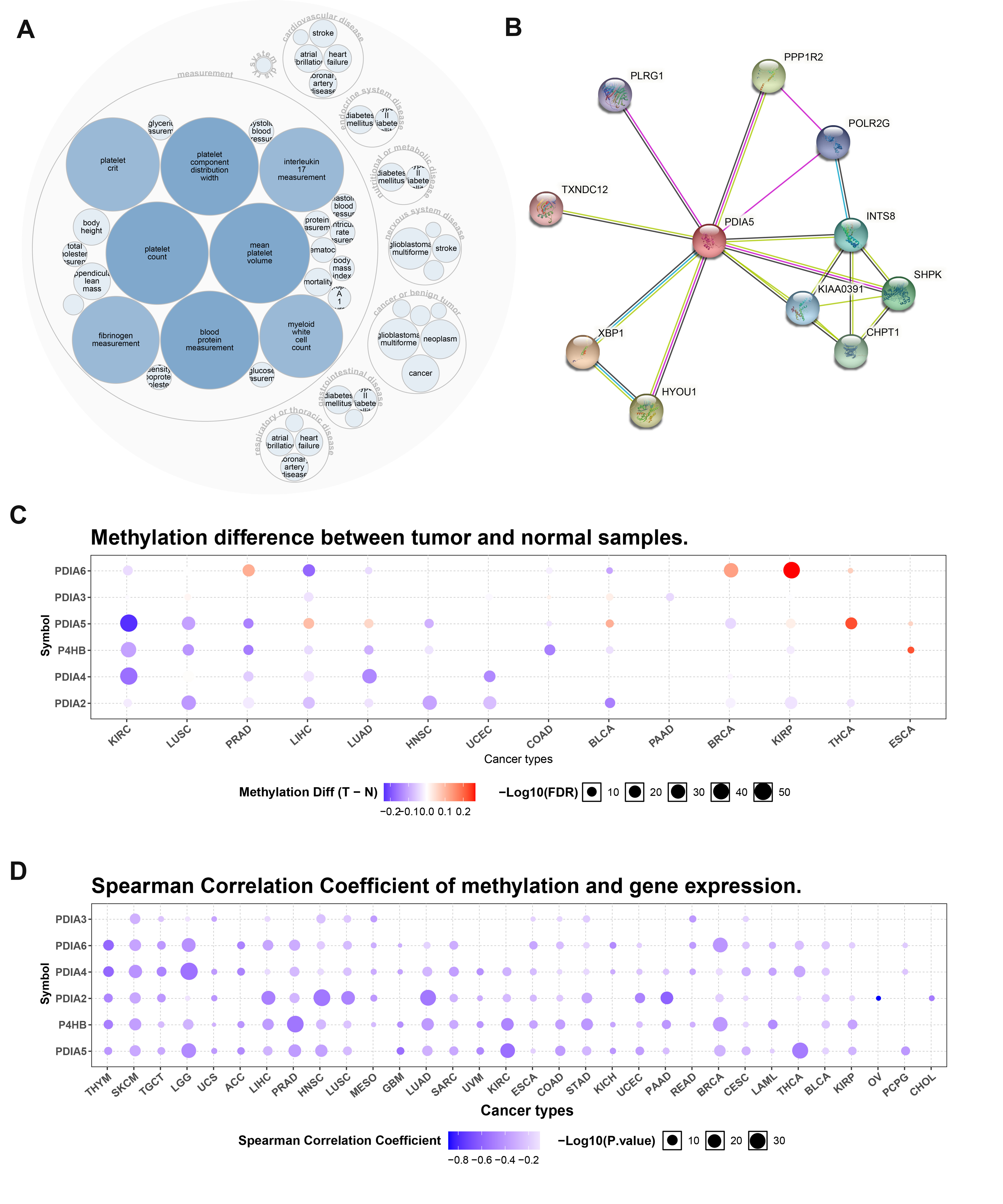

Supplement: Supplementary Figure 1 — The involvement of PDIA5 in human disease (A) and the prediction of the protein-protein interaction network of PDIA5 (B). (C) The methylation difference of PDIA5 and other PDI family genes between tumor and corresponding normal tissues. (D) The relationships between methylation and the gene expression of PDIA5 and other PDI family genes. [file Image_1.jpeg]

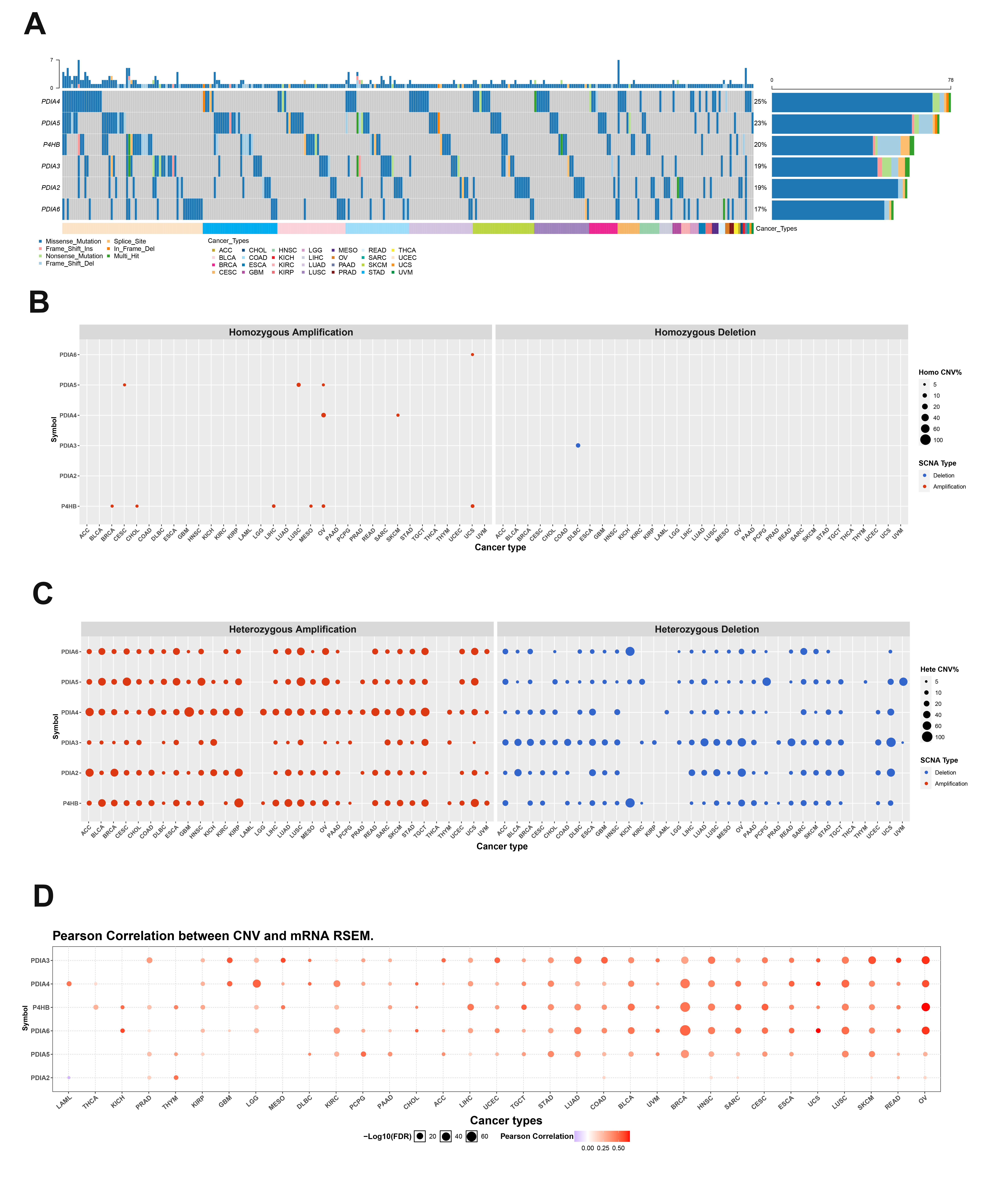

Supplement: Supplementary Figure 2 — Pan-cancer genetic alterations of PDIA5 and other PDI genes. (A) The single nucleotide variation (SNV) frequency of PDIA5 and other PDI genes across 28 cancer types. (B) The frequency of homozygous amplification (left) and deletion (right) of PDIA5 and other PDI genes across cancer types. (C) The frequency of heterozygous amplification (left) and deletion (right) of PDIA5 and other PDI genes across cancer types. (D) The correlation analysis between copy number variations (CNVs) and mRNA expression of PDIA5. [file Image_2.jpg]

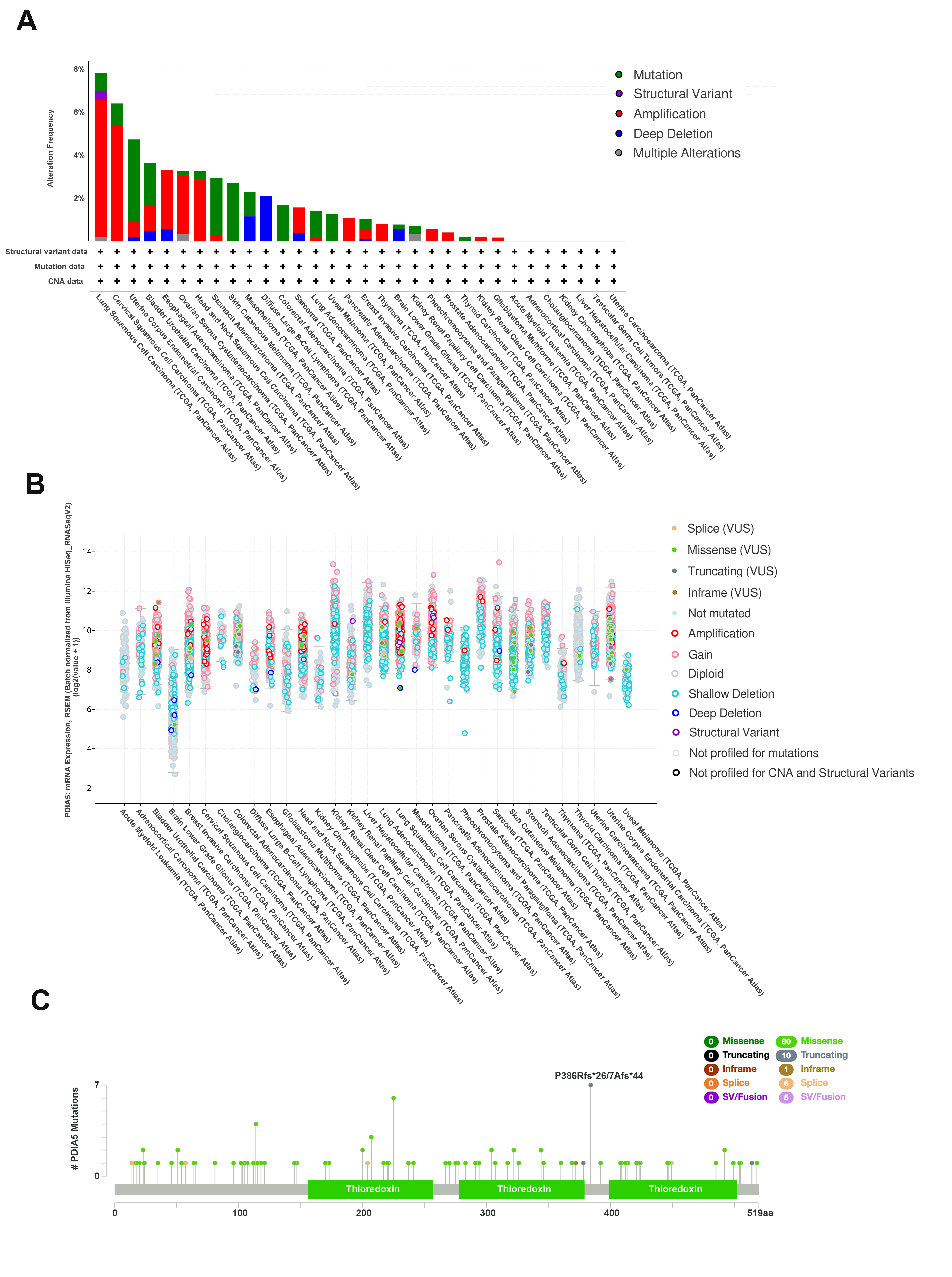

Supplement: Supplementary Figure 3 — The mutation landscape of PDIA5. (A) The mutation frequency of PDIA5 in various cancer using cBioPortal database. (B) The general mutation count of PDIA5 in multiple cancers. (C) The mutation diagram of PDIA5 across protein domains. [file Image_3.jpeg]

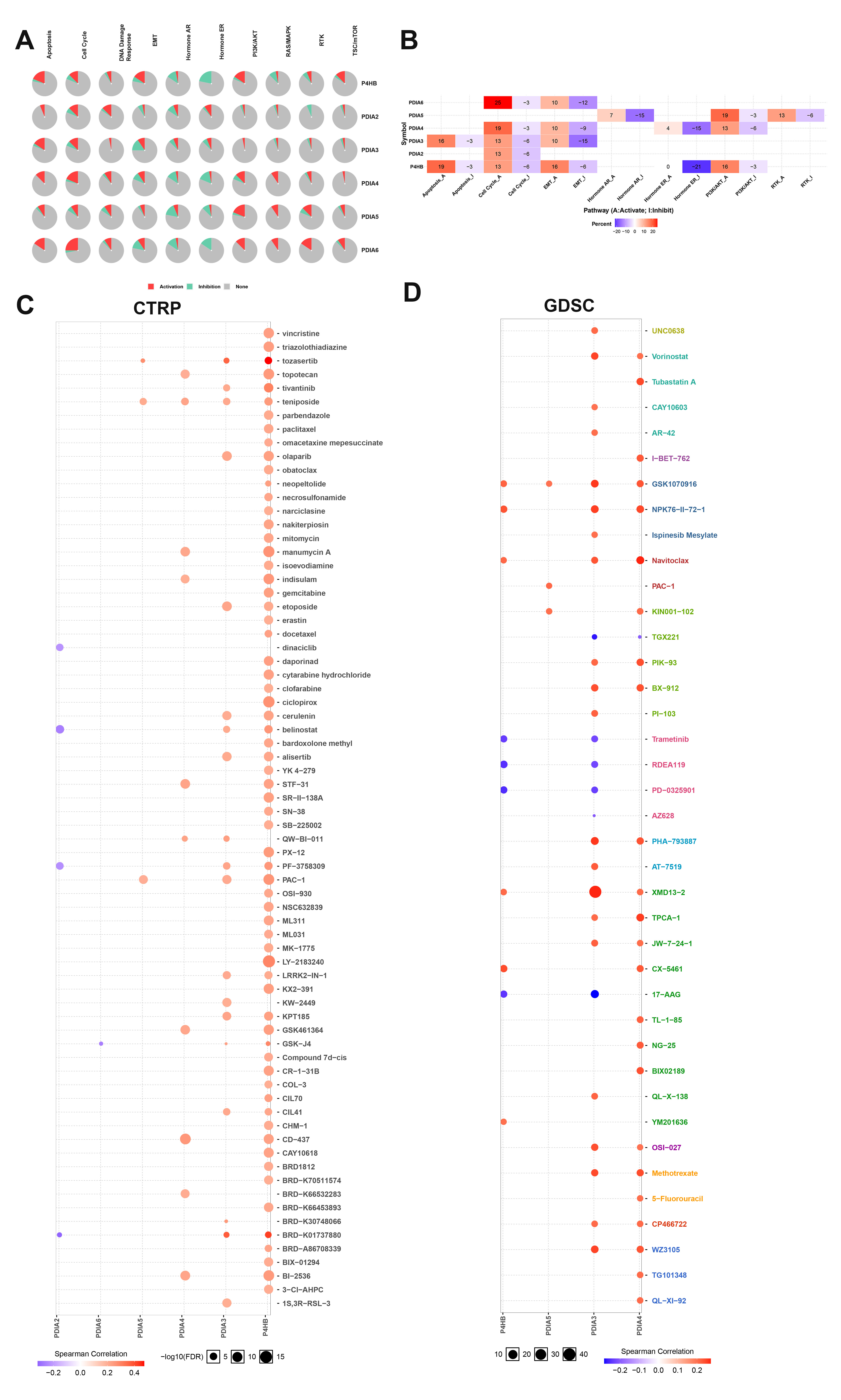

Supplement: Supplementary Figure 4 — The pathway analysis and drug sensitivity of PDIA5 in human cancers. (A, B) The pie chart and network diagram demonstrating the correlations between PDIA5 and cancer pathways. (C, D) The correlation analysis of PDIA5 and IC50 level for each drug in cancer cell lines from the CTRP and GDSC databases. [file Image_4.jpeg]

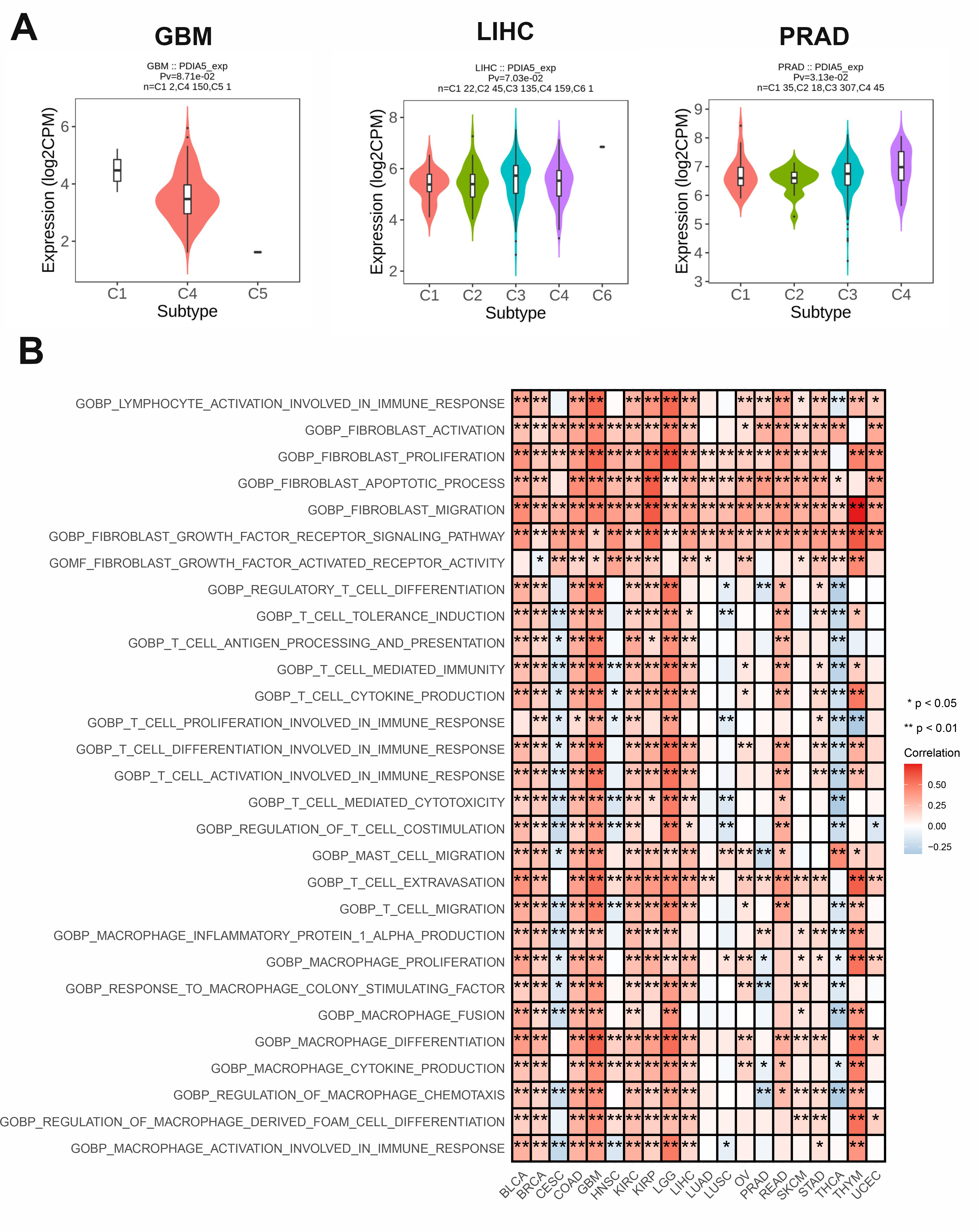

Supplement: Supplementary Figure 5 — PDIA5 is relevant to immune subtypes and immune pathways in human cancers. (A) The relationships between PDIA5 and immune subtypes in GBM, liver hepatocellular carcinoma (LIHC), and PRAD. C1 to C6 represent wound healing, IFN-γ dominant, inflammatory, lymphocyte depleted, immunologically quiet, and TGF-β dominant, respectively. (B) The correlations between PDIA5 and immunity pathways. [file Image_5.jpeg]

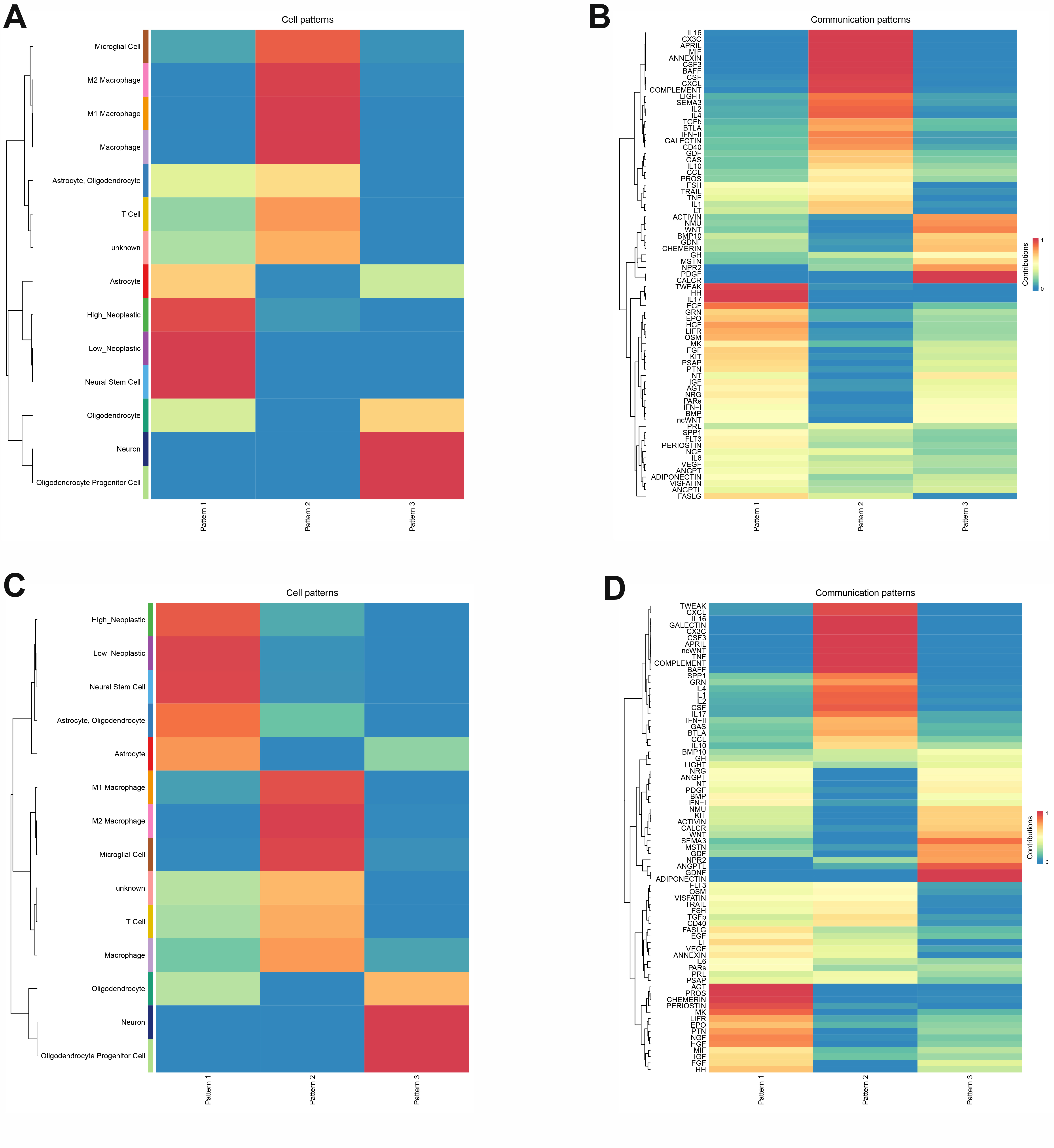

Supplement: Supplementary Figure 9 — The cellular communication of the 14 cell types in GBM. (A, B) The cell patterns and communication patterns with regard to the receiver of the 14 cell types. (C, D) The cell patterns and communication patterns with regard to the sender of the 14 cell types. [file Image_9.jpeg]

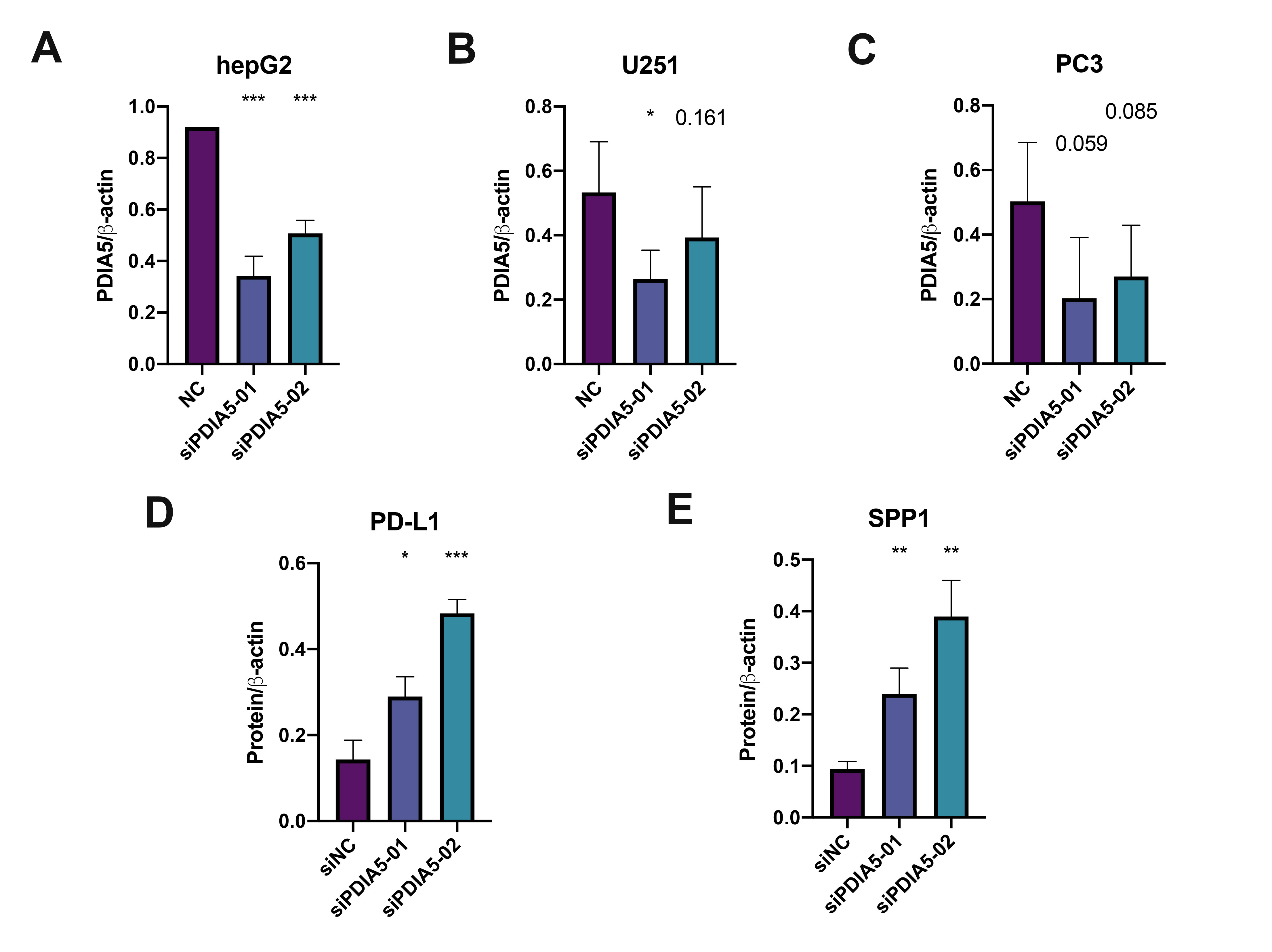

Supplement: Supplementary Figure 12 — PDIA5 regulates the expression of PD-L1 and SPP1 in U251 cells. (A–C) The bar graph showing the protein expression level of PDIA5 in hepG2, U251, and PC3 cells after transfection with siRNA-PDIA5-1 and siRNA-PDIA5-2, respectively. (D) The bar graph displaying the protein expression level of PD-L1 in U251 cells after transfection with siRNA-PDIA5-1 and siRNA-PDIA5-2. (E) The bar graph showing the protein expression level of SPP1 in U251 cells after transfection with siRNA-PDIA5-1 and siRNA-PDIA5-2. Data are displayed as mean ± SD based on three independent experiments. ∗p < 0.05, ∗∗p < 0.01. [file Image_12.jpeg]
